# Sunstroke—How to Treat It

**Published:** 1892-05

**Authors:** 


### SUNSTROKE—HOW TO TREAT IT.

It is the general impression that sunstroke prevails most at the very hottest season. Physicians say it depends rather upon the fact of one's being accustomed to the heat. A very hot spell in June may be worse in its results than the hottest days of August, nor is the brain heating known as sunstroke confined to those exposed to the direct rays of the sun. It is the same thing when a man is overcome by heat in a close room or workshop. From an article by a physician of high standing we gather some important facts and hints as to treatment.

In fatal cases it is found that the brain and lungs are gorged with blood, and the blood itself is thickened as if its watery constituents had been dissipated by excessive heat. There have been several cases in the Pennsylvania Hospital, says this writer, in which the operation of venesection was resorted to with the object of relieving the internal congestion, and in which the blood was so thick that it would not run out and had to be squeezed out by pressure and friction.

For those who are obliged to be in the sun it is advisable to wear light clothing, a straw or felt hat, perforated to allow a free circulation of air ; drink freely of cold water and other refreshing liquids, and aim to drink a small quantity very often rather than a large quantity at once. The large amount of icy fluid entering the stomach at one time chills its surface, and forces three or four ounces of blood to the brain in excess of its natural requirements, resulting in cerebral congestion and death.

Those exposed to the sun ought to bathe the face and head several times during the day. Especially little children ought to be cooled off in this manner. A man who suddenly finds he has stopped perspiring during a hot day is in great danger, especially if his skin feels exceedingly hot. He ought to stop work, get into the shade, and bathe his head with very cold water, and drink either a cup of hot tea or milk, the object being to draw the blood from the brain by a cold effusion to the head and a gentle stimulating drink to the stomach.

In more violent cases, where the man suddenly drops, becomes unconscious, treatment ought to begin at once. The man should be placed in the shade, ice water should be poured on his head or kept against his head by means of cloths, and ordinary cold hydrant or well water poured over his body until a physician arrives, who should be sent for at once.

Sunstroke ought never to be regarded as of slight importance or be neglected for any reason. The mortality is usually high in sunstroke cases. The hospital records give the deaths, in some years, as one-third of those admitted, in spite of all treatment.

